# Oncogenic Role of SATB2 In Vitro: Regulator of Pluripotency, Self-Renewal, and Epithelial–Mesenchymal Transition in Prostate Cancer

**DOI:** 10.3390/cells13110962

**Published:** 2024-06-03

**Authors:** Wei Yu, Rashmi Srivastava, Shivam Srivastava, Yiming Ma, Sharmila Shankar, Rakesh K. Srivastava

**Affiliations:** 1 Kansas City VA Medical Center, 4801 Linwood Boulevard, Kansas City, MO 66128, USAsaowuren@hotmail.com (Y.M.);; 2 Department of Chemistry and Biochemistry, Baylor University, Waco, TX 76798, USA; 3 Louisiana State University, Baton Rouge, LA 70803, USA; 4 John W. Deming Department of Medicine, Tulane University School of Medicine, New Orleans, LA 70112, USA; 5 Southeast Louisiana Veterans Health Care System, 2400 Canal Street, New Orleans, LA 70119, USA; 6 GLAX LLC, 3500 S Dupont Highway, Dover, DE 19901, USA

**Keywords:** SATB2, prostate cancer, cancer stem cells, transformation, Oct4, Sox2, cMyc, Nanog, KLF4

## Abstract

Special AT-rich sequence binding protein-2 (SATB2) is a nuclear matrix protein that binds to nuclear attachment regions and is involved in chromatin remodeling and transcription regulation. In stem cells, it regulates the expression of genes required for maintaining pluripotency and self-renewal and epithelial–mesenchymal transition (EMT). In this study, we examined the oncogenic role of SATB2 in prostate cancer and assessed whether overexpression of SATB2 in human normal prostate epithelial cells (PrECs) induces properties of cancer stem cells (CSCs). The results demonstrate that SATB2 is highly expressed in prostate cancer cell lines and CSCs, but not in PrECs. Overexpression of SATB2 in PrECs induces cellular transformation which was evident by the formation of colonies in soft agar and spheroids in suspension. Overexpression of SATB2 in PrECs also resulted in induction of stem cell markers (CD44 and CD133), pluripotency-maintaining transcription factors (cMYC, OCT4, SOX2, KLF4, and NANOG), CADHERIN switch, and EMT-related transcription factors. Chromatin immunoprecipitation assay demonstrated that SATB2 can directly bind to promoters of BCL-2, BSP, NANOG, MYC, XIAP, KLF4, and HOXA2, suggesting SATB2 is capable of directly regulating pluripotency/self-renewal, cell survival, and proliferation. Since prostate CSCs play a crucial role in cancer initiation, progression, and metastasis, we also examined the effects of SATB2 knockdown on stemness. SATB2 knockdown in prostate CSCs inhibited spheroid formation, cell viability, colony formation, cell motility, migration, and invasion compared to their scrambled control groups. SATB2 knockdown in CSCs also upregulated the expression of E-CADHERIN and inhibited the expression of N-CADHERIN, SNAIL, SLUG, and ZEB1. The expression of SATB2 was significantly higher in prostate adenocarcinoma compared to normal tissues. Overall, our data suggest that SATB2 acts as an oncogenic factor where it is capable of inducing malignant changes in PrECs by inducing CSC characteristics.

## 1. Introduction

Prostate cancer is the second-leading cause of cancer death in American men [1]. It is estimated that in 2024 there will be 299,010 new prostate cancer cases, causing an estimated 35,250 deaths in the United States [1]. Although the mortality rates have been decreasing, in a vast majority of the patients, prostate cancer has metastasized and therefore it becomes incurable. The current clinical treatment mainly includes surgery, chemotherapy, and targeted therapy, but the disease ultimately relapses and is associated with a low 5-year survival of metastatic prostate cancer [2]. The prognosis for men with metastatic prostate cancer is worse, especially due to the development of resistance during treatment [3,4,5]. Prostate cancer initiation, progression, and metastasis are related to many factors such as genetics, lifestyle, and environmental pollution [6,7,8,9,10]. Mounting evidence exists to suggest that CSCs are involved in cancer initiation, promotion, metastasis, and recurrence of the tumor. Therefore, it is essential to understand the contribution of CSCs to prostate carcinogenesis.

The SATB2 (special AT-rich binding protein-2) gene acts as a transcription factor and epigenetic regulator and is involved in chromatin remodeling [11,12,13]. It regulates gene expression both by modulating chromatin architecture and by functioning as a transcriptional co-factor [14,15,16]. This gene is located on chromosome 2q33.1 and is conserved in humans and mice [17]. SATB2 regulates craniofacial patterning and osteoblast differentiation [15], and plays a significant role in the development of cortical neurons [14,16,18,19]. *SATB2* knockout mice are defective in bone development and osteoblast differentiation [15,20]. SATB2 gene is overexpressed in colorectal, breast, lung, liver, neuroendocrine, and pancreatic cancer [21,22,23,24,25,26,27,28,29], and has been associated with cancer progression and poor survival [30]. SATB2 is highly expressed in CSCs and has been shown to regulate the expression of stem cell markers and pluripotency-maintaining factors [22,31,32,33]. Furthermore, the role of SATB2 as a biomarker for certain cancers has been suggested [22,34,35]. However, the oncogenic role of SATB2 in prostate carcinogenesis has never been examined. 

The objective of this paper is to examine the oncogenic role of SATB2 in prostate cancer, and assess whether overexpression of SATB2 in PrECs gained the phenotypes of cancer stem cells (CSCs). Our data showed that SATB2 is highly expressed in human prostate CSCs and cancer cell lines, but not in human normal prostate epithelial cells. Using chromatin immunoprecipitation (ChIP) assay, we have demonstrated that SATB2 can directly bind to the promoters of Nanog, bone sialoprotein (BSP), cMYC, homeobox A2 (HOXA2), B-cell lymphoma 2 (BCL-2), Kruppel-like factor 4 (KLF4), and X-linked inhibitor of apoptosis (XIAP), suggesting SATB2 can act as a regulator of pluripotency and self-renewal of CSCs. Overexpression of SATB2 in PrECs induces malignant transformation, whereas SATB2 knockdown in prostate CSCs inhibits spheroid and colony formation, motility, migration, and invasion. SATB2-transformed PrECs gained the phenotype of CSCs. These data suggest that SATB2 is capable of transforming normal prostate epithelial cells in vitro.

## 2. Materials and Methods

### 2.1. Cell Culture Conditions and Reagents

Prostate cancer cell lines (C4-2B, DU-145, LNCaP, and PC-3) and human normal prostate epithelial cells (PrEC) were purchased from the American Type Culture Collection (ATCC), Manassas, VA, USA. Prostate cancer cell lines were grown in Dulbecco’s Modified Eagle’s Medium with 10% fetal bovine serum with antibiotics. Human prostate CSCs were obtained from Celprogen (Torrance, CA, USA) and grown in a well-defined cell culture medium. Antibodies against SATB2 and β-actin were purchased from Cell Signaling Technology, Inc. (Danvers, MA, USA). Enhanced chemiluminescence (ECL) Western blot detection reagents were purchased from Amersham Life Sciences Inc. (Arlington Heights, IL, USA). Human prostate normal and cancer tissues were obtained from the Co-operative Human Tissue Network (CHTN, Western Division, Vanderbilt University Medical Center; Nashville, TN, USA) and US Biomax (Denwood, MD, USA). 

### 2.2. Lentiviral Particle Production and Transduction

The protocol for lentivirus production and transduction has been described elsewhere [32]. In brief, lentivirus was produced by triple transfection of HEK 293T cells. Packaging 293T cells were plated in 10 cm plates at a cell density of 5 × 10^6^ a day before transfection in DMEM containing 10% heat-inactivated fetal bovine serum. 293T cells were transfected with 4 µg of plasmid and 4 µg of lentiviral vector using lipid transfection (Lipofectamine-2000/Plus reagent, Invitrogen, Waltham, MA, USA) according to the manufacturer’s protocol. Viral supernatants were collected and concentrated by adding PEG-it virus precipitation solution (SBI System Biosciences, Palo Alto, CA, USA) to produce virus stocks with titers of 1 × 10^8^ to 1 × 10^9^ infectious units per mL. Viral supernatant was collected for 3 days by ultracentrifugation and concentrated 100-fold. Titers were determined on 293T cells. Cells were transduced with lentiviral particles expressing the gene of interest. 

### 2.3. Spheroid Assay

Spheroid formation assays were performed as described elsewhere [36]. Briefly, CSCs were plated in six-well ultra-low attachment plates (Corning Inc., Corning, NY, USA) at a density of 100 to 500 cells/mL in stem cell growth medium with 1% N2 Supplement (Invitrogen), 2% B27 Supplement (Invitrogen), 20 ng/mL human platelet growth factor (Sigma-Aldrich, St. Louis, MO, USA), 100 ng/mL epidermal growth factor (Invitrogen), and 1% antibiotic-antimycotic (Invitrogen) at 37 °C in a humidified atmosphere of 95% air and 5% CO_2_. Spheroids were collected after 7 days and dissociated with Accutase (Innovative Cell Technologies, Inc., San Diego, CA, USA). The CSCs obtained from dissociation were counted using trypan blue dye.

### 2.4. Motility Assay

We used scratch motility assay to monitor the horizontal movement of cells as described elsewhere [37]. A monolayer of cells was established and then a scratch was made through the monolayer which gave rise to an in vitro wound. Wells were washed twice with PBS and replaced with fresh medium. The movement of cells to the scratch area as single cells from the confluent sides was monitored. Three replicate wells from a six-well plate were used for each experimental condition.

### 2.5. Transwell Migration Assay

Transwell migration assay was performed as described elsewhere [37]. In brief, 1 × 10^5^ prostate cancer cells were plated in the top chamber onto the noncoated membrane (24-well insert; pore size, 8 μm; Corning Costar, Corning, NY, USA) and allowed to migrate toward serum-containing medium in the lower chamber. Cells were fixed after 48 h of incubation with methanol and stained with 0.1% crystal violet (2 mg/mL, Sigma-Aldrich). The number of cells invading through the membrane was counted under a light microscope.

### 2.6. Transwell Invasion Assay

Transwell invasion assay was performed as described elsewhere [33]. In brief, 1 × 10^5^ cells were plated in the top chamber onto the Matrigel-coated membrane (24-well insert; pore size, 8 μm; Corning Costar). Each well was coated freshly with Matrigel (60 μg; BD Bioscience, Franklin Lakes, NJ, USA) before the invasion assay. Cancer cells were plated in a medium without serum or growth factors, and a medium supplemented with serum was used as a chemoattractant in the lower chamber. The cells were incubated for 72 h and cells that did not invade through the pores were removed using a cotton swab. Cells on the lower surface of the membrane were fixed with methanol and stained with crystal violet. The number of cells invading through the membrane was counted under a light microscope.

### 2.7. Western Blot Analysis

Western blot analysis was performed as we described earlier [38]. In brief, cell lysates were subjected to SDS-PAGE, and gels were blotted on the nitrocellulose membrane (Amersham Biosciences, Piscataway, NJ, USA). The membranes were blocked with 5% skim milk or 5% BSA in Tris-Tween buffered saline at 37 °C for 2 h and then blotted with primary antibody diluted in Tris-buffered saline (1:1000 dilutions) overnight at 4 °C. The membranes were then washed three times with tris-buffered saline-T (TBS-T) and incubated with a secondary antibody linked to horseradish peroxidase (1:5000) for 1 h. After incubation with a secondary antibody, the membranes were washed again three times with TBS-T. Finally, protein antibody complexes were detected by the addition of ECL substrate (Thermo Fisher Scientific, Rockford, IL, USA).

### 2.8. Chromatin Immunoprecipitation (ChIP) Assay

Prostate CSCs were fixed with 1% formaldehyde for 15 min (RT), quenched with 125 mM glycine for 5 min (RT), centrifuged and resuspended in RIPA buffer containing protease inhibitors, and incubated on ice (10 min). Samples were sonicated (Heat Systems-Ultrasonic device) to shear chromatin to an average length of about 1 Kb and transferred to 1.5 mL tubes, then microcentrifuged for 10 min (max speed). Supernatants were collected in 1.5 mL tubes containing 1 mL of the dilution buffer (0.01% SDS, 1.1% Triton, 1.2 mM EDTA, 167 mM NaCl, 17 mM Tris, pH 8). 3 μg of SATB2 antibody was added to the samples, and samples were incubated overnight at 4 °C, followed by the addition of 5 μL of protein-A and protein-G magnetic beads (Invitrogen) for 2 h. Beads were collected with a magnet (Thermo), washed 4× with 1 mL of each of four wash buffers (Wash Buffer 1: 0.1% SDS, 1% Triton, 2 mM EDTA, 150 mM NaCl, 20 mM Tris, pH 8; Wash Buffer 2: 0.1% SDS, 1% Triton, 2 mM EDTA, 500 mM NaCl, 20 mM Tris, pH 8; Wash Buffer 3: 0.25 M LiCl, 1% NP-40, 1% deoxycholate, 1 mM EDTA, 10 mM Tris, pH 8; Wash Buffer 4: 10 mM Tris, pH 8, 1 mM EDTA). After the last wash, 50 μL of a 10% Chelex-100 (Bio-Rad, Hercules, CA, USA) resin solution was added to the beads, samples were boiled (10 min) in a heat block, microcentrifuged (1 min, max speed), supernatants were collected followed by the addition of 50 μL of MQ water back to the beads, microcentrifuged again (1 min, max speed), and the new supernatant pooled with the previous one. An amount of 1–3 µL elutions were used for PCR reaction.

### 2.9. Quantitative Real-Time PCR

Total RNA was isolated using an RNeasy Mini Kit (Qiagen, Valencia, CA, USA). Briefly, cDNA was synthesized using a high-capacity cDNA reverse transcription kit (Applied Biosystems, Waltham, MA, USA). Primers specific for each of the signaling molecules were designed using NCBI/Primer-BLAST and used to generate the PCR products. For the quantification of gene amplification, real-time PCR was performed using an ABI 7300 Sequence Detection System in the presence of SYBR- Green. Target sequences were amplified at 95 °C for 10 min, followed by 40 cycles of 95 °C for 15 s and 60 °C for 1 min. HK-GAPD was used as an endogenous normalization control. All assays were performed in triplicate and were calculated based on the ΔΔCt method. The n-fold change in mRNA expression was determined according to the method of 2^−ΔΔCT^.

### 2.10. Immunocytochemistry

For immunocytochemistry, cells were grown on fibronectin-coated coverslips (Becton Dickinson, Bedford, MA, USA). After 24 h, cells were fixed with methanol, permeabilized with 1% NP-40, and blocked with 10% BSA, followed by incubating with SATB2 antibody (1:100 dilution). After washing, cells were incubated with FITC-labeled secondary antibody (1:1000 dilution) and 4′,6-diamidino-2-phenylindole (DAPI, 1 µg/mL). Finally, coverslips were washed and mounted using Vectashield (Vector Laboratories, Burlington, CA, USA). Stained slides were examined under a fluorescence microscope. For a better visualization, the color of the nucleus was changed from blue to red. 

### 2.11. Immunohistochemical (IHC) Staining 

Prostate adjacent normal and cancerous (adenocarcinoma) tissues were purchased from US Biomax (Denwood, MD, USA) and Cooperative Human Tissue Network (CHTN). Tissue sections were incubated with SATB2 primary antibody (1:100) at 4 °C overnight, then washed with phosphate-buffered saline (PBS) three times, and incubated with horseradish peroxidase (HRP) conjugated goat anti-mouse IgG polyclonal antibody for 30 min at room temperature. A goat anti-mouse HRP-polymer detection kit was used to stain the tissue slides. Photographs of prostate normal and cancerous tissues were obtained by microscopy. The intensity of staining was graded on a scale of 0–3, according to the following assessment: no detectable stating (0), weak staining (1), moderate staining (2), and strong staining (3). IHC scoring (H-score) was calculated as follows: (0 × percent of no detectable staining) + (1 × percent of weak staining) + (2 × percent of moderate staining) + (3 × percent of strong staining). The receiver operating characteristic (ROC) curve of prostatic tissues was also plotted.

### 2.12. Statistical Analysis

The mean and SD were calculated for each experimental group with replicates. Differences between groups were analyzed by ANOVA, followed by Bonferoni’s multiple comparison tests using PRISM statistical analysis software (GrafPad Software, Inc., San Diego, CA, USA). Significant differences among groups were calculated at *p* < 0.05.

## 3. Results

### 3.1. SATB2 Is Not Expressed in Human Normal Prostate Epithelial Cells, but It Is Highly Expressed in Prostate Cancer Cell Lines

**Figure 1:**
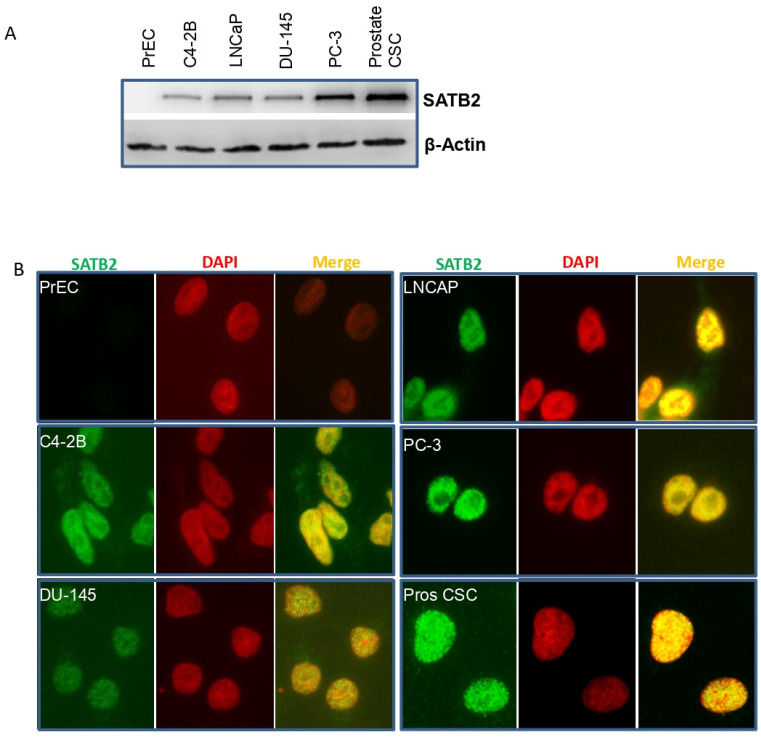
The expression of SATB2 in human normal prostate epithelial cells, prostate cancer cell lines, and prostate CSCs. (**A**) Expression of SATB2 protein. Crude proteins from human prostate normal epithelial cells (PrEC), prostate cancer cell lines (C4-2B, LNCaP, DU-145, and PC-3), and prostate CSCs were isolated and the expression of SATB2 was measured by Western blot analysis. β-actin was used as a loading control. (**B**) Expression of SATB2 by immunocytochemistry (ICC). Human prostate normal epithelial cells (PrEC), prostate cancer cell lines (C4-2B, LNCaP, DU-145, and PC-3), and prostate CSCs were grown and the expression of SATB2 was measured by immunocytochemistry. Magnification 40×.

We first compared the expression of SATB2 in human normal prostate epithelial cells (PrEC), prostate cancer cell lines (C4-2B, LNCaP, DU-145, and PC-3), and prostate CSCs using Western blot analysis and immunocytochemistry. As shown in Figure 1A, SATB2 is not expressed in PrECs. However, it is highly expressed in prostate cancer C4-2B, LNCaP, DU-145, and PC-3 cell lines, and prostate CSCs. Interestingly, the expression of SATB2 was highest in CSCs. A similar expression pattern of SATB2 was observed by immunocytochemistry (Figure 1B). These data suggest that the expression of SATB2 is tightly regulated in prostate cancer cells and it may have a role in prostate malignancy.

### 3.2. Overexpression of SATB2 in Human Normal Prostate Epithelial (PrEC) Cells Induces Cellular Transformation and Stemness

**Figure 2:**
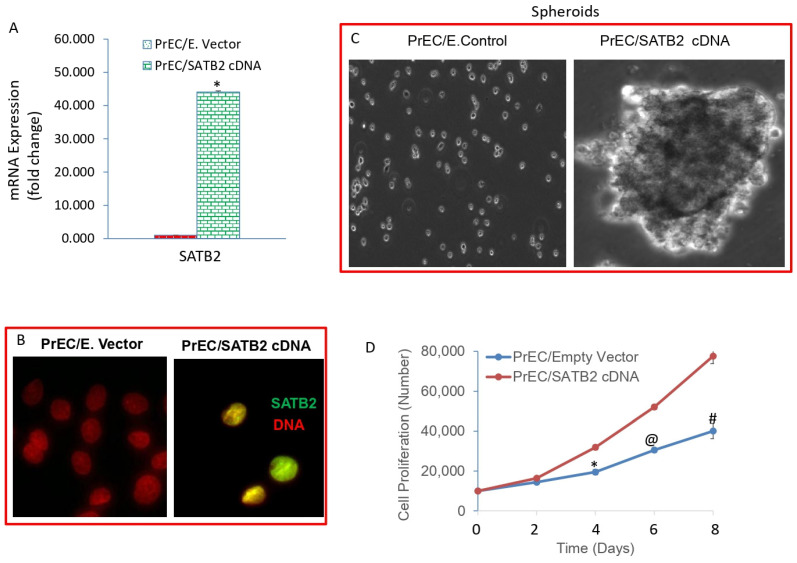
Overexpression of SATB2 in PrECs results in spheroid formation and enhances cell proliferation. (**A**) Expression of SATB2 mRNA. PrECs were transduced with lentiviral particles expressing either Empty Vector or SATB2 cDNA. SATB2 expression was measured by the qRT-PCR. Data represent mean (n = 4) ± SD. * = significantly different from the respective Empty Vector group, *p* < 0.05. (**B**) Expression of SATB2 protein by immunocytochemistry. PrECs were transduced with lentiviral particles expressing either Empty Vector or SATB2 cDNA. SATB2 expression was measured by immunocytochemistry. Note: No expression of SATB2 protein was detected in PrEC/Empty Vector group. (**C**) Spheroid formation in suspension. PrEC/Empty Vector and PrEC/SATB2 cells were grown in suspension for one week in an ultra-low attachment plate in a defined stem cell culture medium. Spheroid pictures were taken with a microscope. (**D**) Cell proliferation of PrEC/Empty Vector and PrEC/SATB2 groups was measured for 8 days. Data represent mean (n = 4) ± SD. *, @, and # = significantly different from respective Empty Vector group, *p* < 0.05. Magnification 40×.

During cellular transformation, genetic and phenotypic changes occur in cells [39,40,41]. Some common cellular features such as high/indefinite saturation density, lack of contact inhibition, less oriented growth, loss of tight junction, and the formation of colonies have been observed in transformed cells. To prove that SATB2 induces cellular transformation and stemness, we overexpressed SATB2 in prostate normal epithelial cells (PrECs). Lentiviral-mediated infection of SATB2 gene in PrECs (PrEC/SATB2) resulted in an increased expression of SATB2 mRNA (Figure 2A). Similarly, immunocytochemistry data confirmed that SATB2 was highly expressed in PrEC/SATB2 cells (Figure 2B). The expression of SATB2 was not observed in the PrEC/Empty Vector group. 

We next examined whether SATB2 induces cellular transformation. Overexpression of SATB2 induces cellular transformation as evidenced by formation of spheroids in suspension (Figure 2C, right panel). Normal PrECs (PrEC/Empty Vector) fail to form spheroids in suspension (Figure 2C, left panel). Furthermore, PrEC/SATB2 cells demonstrated enhanced cell growth compared to PrEC/Empty Vector cells (Figure 2D). Overall, these data suggest that SATB2 alone is capable of inducing transformation of PrECs in vitro, and may have a role in prostate cancer initiation.

**Figure 3:**
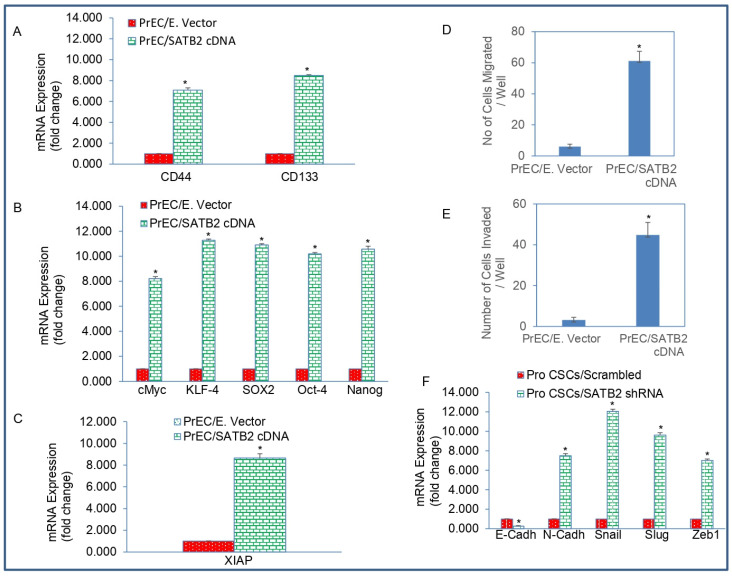
Overexpression of SATB2 in PrECs induces stemness. (**A**) RNA was isolated and the expression of stem cell marker CD44 and CD133 was measured by qRT-PCR analysis. Data represent mean (n = 4) ± SD. * = significantly different from PrEC/Empty Vector group (*p* < 0.05). (**B**) Expression of pluripotency-maintaining factors. RNA was isolated and the expression of cMYC, KLF4, SOX2, OCT4, and NANOG was measured by qRT-PCR analysis. Data represent mean (n = 4) ± SD. * = significantly different from PrEC/Empty Vector group (*p* < 0.05). (**C**) Expression of XIAP. RNA was isolated and the expression of XIAP was measured by qRT-PCR analysis. Data represent mean (n = 4) ± SD. * = significantly different from PrEC/Empty Vector group (*p* < 0.05). (**D**) Transwell migration assay. Transwell migration assay was performed as described in Section 2. Data represent mean ± SD. * = significantly different at *p* < 0.05. (**E**) Transwell invasion assay. Transwell invasion assay was performed as described in Section 2. Data represent mean ± SD. * = significantly different at *p* < 0.05. (**F**) RNA was isolated and the expression of EMT-related genes E-CADHERIN, N-CADHERIN, SNAIL, SLUG, and ZEB1 was measured by qRT-PCR analysis. Data represent mean (n = 4) ± SD. * = significantly different from PrEC/Empty Vector group (*p* < 0.05). Gene expression of Empty Vector was normalized to 1.

We next measured whether the SATB2-transformed cells gained the properties of stemness by expressing stem cell markers and pluripotency-maintaining factors. Overexpression of SATB2 in PrECs resulted in the induction of CD44 and CD133 genes (Figure 3A). Since SATB2 induced stem cell markers, we next examined the expression of pluripotency-maintaining transcription factors. Overexpression of SATB2 in PrECs resulted in induction of cMYC, KLF4, SOX2, OCT4, and NANOG genes (Figure 3B). These data suggest that overexpression of SATB2 is capable of transforming cells by inducing stem cell markers and pluripotency-maintaining factors.

The expression of X-linked inhibitor of apoptosis (XIAP) has been documented in stem cells [42]. We therefore sought to examine whether overexpression of SATB2 in prostate epithelial cells upregulates XIAP. Overexpression of SATB2 in PrECs resulted in upregulation of XIAP (Figure 3C). These data suggest that overexpression of SATB2 is capable of upregulating XIAP. 

Epithelial to mesenchymal transition (EMT) is a process whereby epithelial cells undergo transition to a mesenchymal phenotype and contribute directly to stemness and cancer cell metastasis [43]. We therefore sought to examine whether overexpression of SATB2 in normal PrECs enhances cell migration and invasion. Overexpression of SATB2 in normal PrECs enhances cell migration and invasion (Figure 3D,E).

Since overexpression of the SATB2 gene induces cell migration and invasion, we next sought to examine the effects of SATB2 on the expression of EMT-related genes (Figure 3F). Overexpression of SATB2 in PrECs (PrEC/SATB2 group) inhibited the expression of E-CADHERIN, and upregulated the expression of N-CADHERIN and transcription factors SNAIL, SLUG and ZEB1. These data suggest that the SATB2 gene is capable of inducing EMT by modulating the expression of CADHERINS and EMT-related transcription factors. Overall, these data suggest that SATB2 can induce cellular transformation in PrECs by inducing CSCs/progenitor cells which are capable of expressing stem cell markers, transcription factors, and EMT-related genes. 

### 3.3. Knockdown of SATB2 in Prostate CSCs Inhibits Spheroid Formation, and Markers of Pluripotency, Stem Cells, and Cell Survival/Proliferation

**Figure 4:**
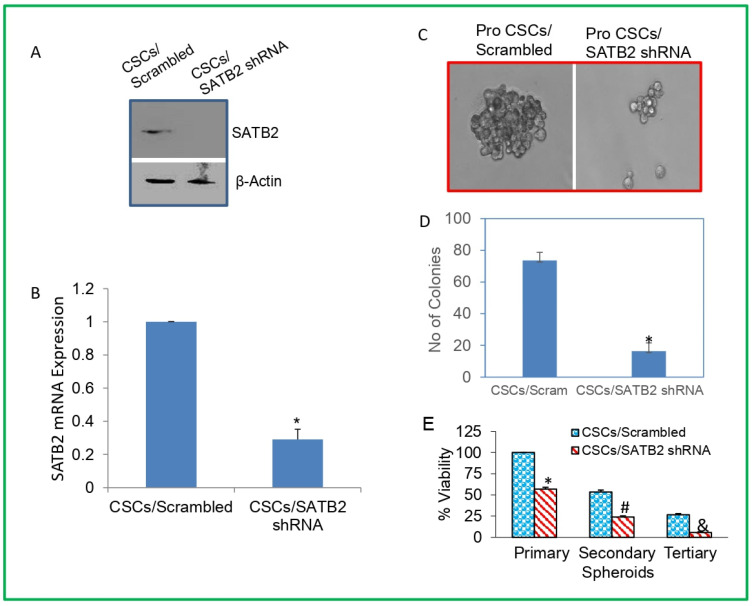
SATB2 knockdown in CSCs inhibits spheroid and colony formation, and cell viability. (**A**) Prostate CSCs were transduced with lentiviral particles expressing either scrambled or SATB2 shRNA. Cell lysates were prepared and protein expression of SATB2 was measured by Western blot analysis. β-actin was used as a loading control. (**B**) mRNA expression. RNA was isolated from CSCs/Scrambled and CSCs/SATB2 shRNA groups. qRT-PCR was performed to measure the expression of mRNA. Data represent mean ± SD. * = significantly different at *p* < 0.05. (**C**) Spheroid formation. Spheroids in suspension were generated from CSCs/Scrambled and CSCs/SATB2 shRNA groups. Representative photographs (40×) of spheroids were taken from cell cultures at day 7. (**D**) Colony formation. Prostate CSCs/Scrambled and Prostate CSCs/SATB2 shRNA cells were grown for 21 days, and the number of colonies were counted. Data represent mean (n = 4) ± SD. * = significantly different between groups (*p* < 0.05). (**E**) Cell viability in spheroids. Prostate CSCs/Scrambled and Prostate CSCs/SATB2 shRNA cells were seeded on an ultra-low attachment plate in suspension for 7 days to obtain primary spheroids. At the end of the incubation period, spheroids were collected and reseeded for another week to obtain secondary spheroids. At the end of the second incubation period (two weeks), spheroids were collected and reseeded for another week to obtain tertiary spheroids. Cell viability in spheroids was measured by trypan blue assay at the end of 7, 14, and 21 days. Data represent mean ± SD. *, #, and & = significantly different from control, *p* < 0.05.

Prostate CSCs are capable of cancer initiation, progression, and metastasis, and they express stem cell markers and pluripotency-maintaining factors [44,45,46,47]. We therefore sought to examine whether inhibition of SATB2 in prostate CSCs by shRNA technology inhibits stem cell properties. We examined the effects of inhibiting SATB2 by shRNA in CSCs on spheroid formation, colony formation, cell viability, and markers of stem cells and pluripotency/self-renewal. We completed the transduction of prostate CSCs with lentiviral particles expressing SATB2 shRNA inhibited protein and mRNA expression of SATB2 (Figure 4A,B). We next examined the effects of inhibiting SATB2 on spheroid formation. SATB2 knockdown in prostate CSCs inhibited spheroid formation (Figure 4C).

We next examined the effects of SATB2 knockdown on colony formation and cell viability. We completed the transduction of prostate CSCs with lentiviral particles expressing SATB2 shRNA inhibited colony formation (Figure 4D) and cell viability of primary, secondary and tertiary spheroids compared to a scrambled control group (Figure 4E).

**Figure 5:**
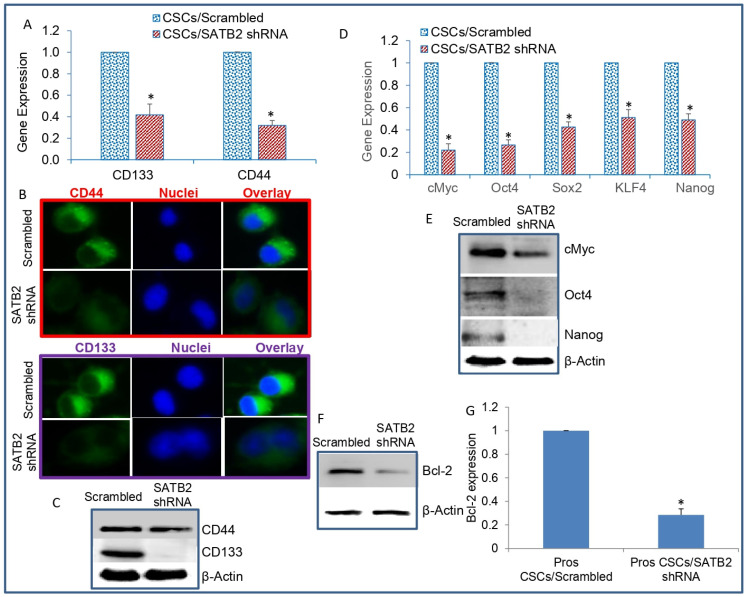
SATB2 shRNA inhibits markers of stem cells, pluripotency/self-renewal, and cell survival/proliferation. (**A**) Expression of stem cell markers. Prostate CSCs were transduced with lentiviral particles expressing either scrambled or SATB2 shRNA. RNA was isolated and the expression of CD44 and CD133 was measured by qRT-PCR. GAPDH was used as an internal control. Data represent mean (n = 4) ± SD. * = significantly different between groups (*p* < 0.05). (**B**) Immunohistochemistry of CD44 and CD133 (40×). Prostate CSCs/Scrambled and Prostate CSCs/SATB2 shRNA cells were stained with either an anti-CD44 antibody or an anti-CD133 antibody. Expression of CD44 and CD133 was examined as described in Section 2. (**C**) Protein expression of CD44 and CD133. Cell lysates were collected from Prostate CSCs/Scrambled and CSCs/SATB2 shRNA cells. The expression of CD44 and CD133 was measured by Western blot analysis. β-actin was used as a loading control. (**D**) Expression of pluripotency/self-renewal factors. RNA was isolated from Prostate CSCs/Scrambled and CSCs/SATB2 shRNA cells, and the expression of cMYC, OCT4, SOX2, KLF4, and NANOG was measured by qRT-PCR. GAPDH was used as an internal control. Data represent mean (n = 4) ± SD. * = significantly different between groups (*p* < 0.05). (**E**) Protein expression of cMYC, OCT4, and NANOG. Crude proteins were isolated from Prostate CSCs/Scrambled and Prostate CSCs/SATB2 shRNA cells. The expression of cMYC, OCT4, and NANOG was measured by Western blot analysis. β-actin was used as a loading control. (**F**) Protein expression of BCL-2. Crude proteins were isolated from Prostate CSCs/Scrambled and Prostate CSCs/SATB2 shRNA cells. The expression of BLC-2 was measured by Western blot analysis. β-actin was used as a loading control. (**G**) RNA expression of BCL-2. RNA was isolated from Prostate CSCs/Scrambled and Prostate CSCs/SATB2 shRNA cells and the expression of BCL-2 was measured by qRT-PCR. GAPDH was used as an internal control. Data represent mean (n = 4) ± SD. * = significantly different between groups (*p* < 0.05).

We next examined the mechanisms by which SATB2 knockdown inhibited the growth of CSCs by measuring stem cell markers and pluripotency-maintaining factors. SATB2 knockdown inhibited the mRNA expression of CD44 and CD133 as measured by qRT-PCR (Figure 5A). We next confirmed the expression of CD44 and CD133 by immunocytochemistry and Western blot analysis. SATB2 knockdown inhibited the protein expression of CD44 and CD133 (Figure 5B,C). We next measured the effects of SATB2 knockdown on pluripotency-maintaining factors by qRT-PCR. SATB2 knockdown inhibited the mRNA expression of cMYC, OCT4, SOX2, KLF4, and NANOG (Figure 5D). Similarly, SATB2 knockdown inhibited the protein expression of cMYC, OCT4, and NANOG (Figure 5E). These data suggest that SATB2 can regulate stemness by modulating the expression of pluripotency-maintaining factors. 

We next examined whether inhibition of SATB2 regulates BCL-2 expression, and regulators of cell proliferation, survival, and apoptosis. As shown in Figure 5F,G, SATB2 knockdown inhibited the expression of BCL-2 protein and mRNA in prostate CSCs. Overall, these data suggest that SATB2 can regulate the expression of genes that regulate pluripotency/self-renewal, stem cell markers, and cell survival/proliferation in prostate CSCs.

### 3.4. Knockdown of SATB2 in Prostate CSCs Inhibits Cell Motility, Migration, Invasion, and Markers of EMT

**Figure 6:**
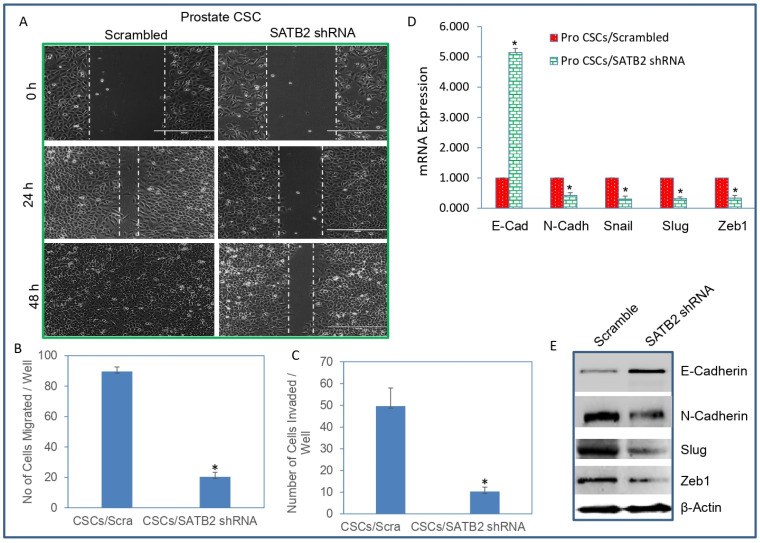
SATB2 shRNA inhibits cell motility, migration, and invasion in prostate CSCs. (**A**) Cell motility assay (40×). Prostate CSCs/Scrambled and CSCs/SATB2 shRNA cells were grown in petri dishes. After 18 h of incubation, cells were scratched with the fine pipette tips. Phase contrast images of scratched cells were captured at 0 h, 24 h, and 48 h time points. (**B**) Transwell migration assay. Transwell migration assay was performed as described in Section 2. Data represent mean ± SD. * = significantly different at *p* < 0.05. (**C**) Transwell invasion assay. Transwell invasion assay was performed as described in Section 2. Data represent mean ± SD. * = significantly different at *p* < 0.05. (**D**) mRNA expression of EMT markers. RNA was isolated and the expression of E-CADHERIN, N-CADHERIN, SNAIL, SLUG, and ZEB1 in Prostate CSCs/Scrambled and CSCs/SATB2 shRNA groups was measured by qRT-PCR. GAPDH was used as an internal control. Data represent mean (n = 4) ± SD. * = significantly different from AsPC-1/Scrambled group (*p* < 0.05). (**E**) Protein expression of EMT markers. The expression of E-CADHERIN, N-CADHERIN, SNAIL, SLUG, and ZEB1 was measured in CSCs/Scrambled and CSCs/SATB2 shRNA cells by Western blot analysis. β-actin was used as a loading control. Data represent mean (n = 4) ± SD.

We next examined whether inhibition of SATB2 in CSCs attenuates cell motility, migration, and invasion of prostate CSCs. Prostate CSCs were transduced with either scrambled or SATB2 shRNA lentiviral particles as described above. SATB2 knockdown inhibited cell motility of CSCs compared to that of the scrambled control in a time-dependent manner (Figure 6A). Similarly, SATB2 knockdown inhibited cell migration and invasion of prostate CSCs compared to the scrambled control group (Figure 6B,C). Since SATB2 knockdown inhibited cell motility, migration, and invasion, we sought to examine the molecular mechanism of these events. As shown in Figure 6D, SATB2 knockdown upregulated the expression of E-CADHERIN and inhibited the expression of N-CADHERIN, SNAIL, SLUG, and ZEB1. We next confirmed the expression of EMT regulators by Western blot analysis. SATB2 knockdown upregulated the protein expression of E-CADHERIN and inhibited the protein expression of N-CADHERIN, SLUG, and ZEB1. These data suggest that inhibition of SATB2 suppresses prostate the motility, migration, and invasion of CSCs and modulates the expression of EMT-related genes.

### 3.5. SATB2 Directly Binds to NANOG, BSP, MYC, HOXA2, BCL-2, KLF4, and XIAP in Prostate Cancer Stem Cells (CSCs)

**Figure 7:**
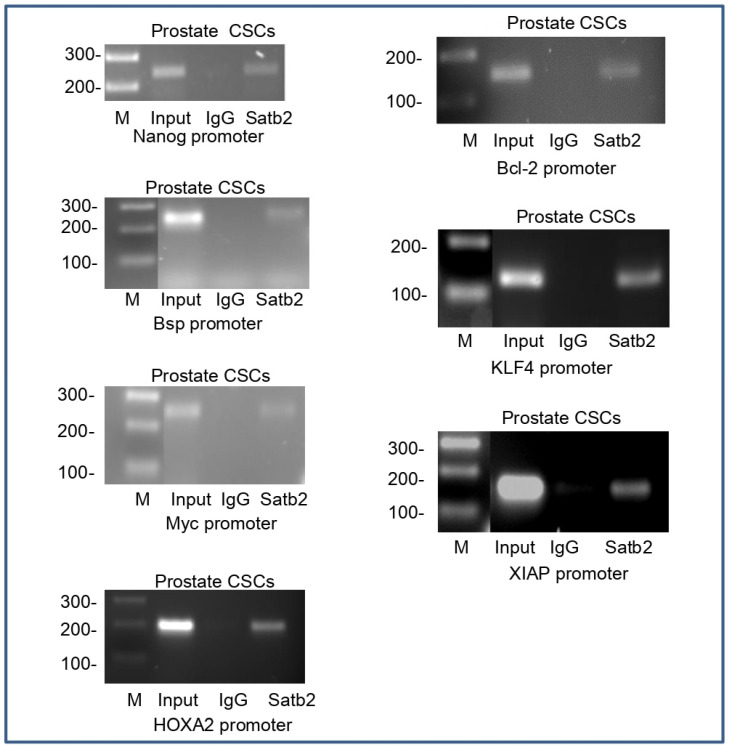
Binding of SATB2 to promoters of NANOG, BSP, MYC, HOXA2, BCL-2, KLF4, and XIAP. Nuclear extracts were prepared from prostate CSCs. Chromatin immunoprecipitation assays were performed to examine the binding of the SATB2 to the promoters of NANOG, BSP, MYC, HOXA2, BCL-2, KLF4, and XIAP in prostate CSCs as described elsewhere [14,16].

We next carried out ChIP assay to identify gene promoters which were a possible candidate for interacting with SATB2. Since SATB2 is a transcription factor, it regulates the expression of genes which play crucial roles in stemness, cell proliferation, and survival [48]. Therefore, we sought to examine whether SATB2 can directly bind to genes such as NANOG, BSP, MYC, HOXA2, BCL-2, KLF4, and XIAP which regulate stemness, cell growth, and survival. As shown in Figure 7, we found that SATB2 can directly bind to promoters of NANOG, BSP, MYC, HOXA2, BCL-2, KLF4, and XIAP. These data suggest that SATB2 can regulate several cellular functions by regulating pluripotency, cell survival, and proliferation genes.

### 3.6. SATB2 Is Highly Expressed in Human Prostate Adenocarcinoma Compared to Normal Prostate Tissues

**Figure 8:**
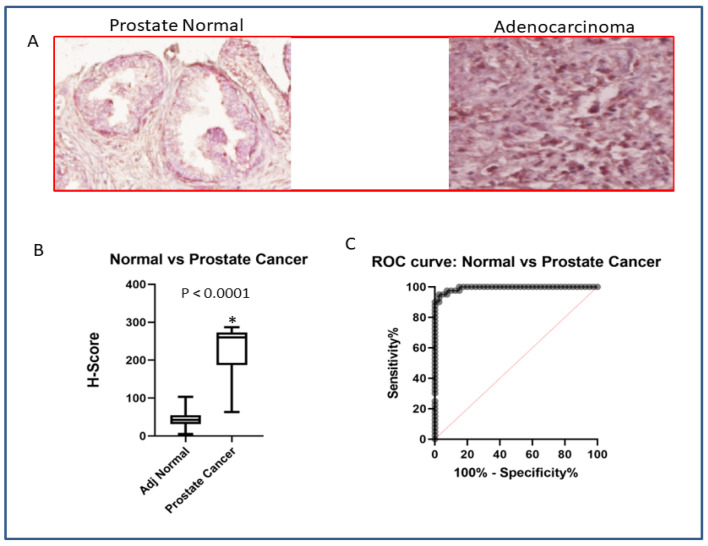
Expression of SATB2 in prostate cancer. (**A**) Expression of SATB2. Prostate tissues containing adjacent normal and cancerous (adenocarcinoma) tissues were purchased from US Biomax (Denwood, MD, USA) and Cooperative Human Tissue Network (CHTN). The expression of SATB2 was measured by immunohistochemistry. Representative photograph of prostate adjacent normal and cancer tissues. Magnification 40×. (**B**) H-score of SATB2 expression in human prostate normal and adenocarcinoma tissues. Data represent mean (n = 40) ± SD. * = significantly different from adjacent normal (*p* < 0.0001). (**C**) ROC curve. ROC curve of prostate normal and adenocarcinoma tissues.

We next compared the expression of SATB2 in human prostate normal and adenocarcinoma tissues by IHC. Expression of SATB2 protein was significantly higher in prostate adenocarcinoma tissues than in normal tissues (Figure 8A,B). Further, the expression of SATB2 was absent or low in adjacent normal tissues. An ROC curve (receiver operating characteristic curve) shows the trade between the true positive fraction and false positive fraction as one changes the criterion for positivity. The ROC curve demonstrated the expression of SATB2 with high sensitivity and specificity (Figure 8C). The area under the ROC curve was 0.9931 ± 0.00549 SE (95% CI = 0.9824 to 1, *p* < 0.0001). Since SATB2 protein is highly expressed in prostate adenocarcinoma tissues, patients are more likely to have an aggressive disease compared to those of a healthy person.

## 4. Discussion

Here, we have shown that overexpression of SATB2 alone can induce the transformation of human normal prostate epithelial cells in vitro, which was evident by the generation of stem cell-like characteristics. These transformed cells expressed stem cell markers CD44 and CD133, and pluripotency-maintaining factors NANOG, OCT4, SOX2, cMYC, and KLF4. Furthermore, SATB2-induced in vitro transformation resulted in the formation of spheroids in suspension and gain of EMT characteristics (downregulation of E-CADHERIN and upregulation of N-CADHERIN expression, and enhanced cell motility, migration, and invasion). SATB2 knockdown in prostate CSCs inhibited stem cell characteristics (spheroid formation, expression of stem cell markers and pluripotency-maintaining factors, and EMT-related genes). Chromatin immunoprecipitation assay demonstrated that SATB2 can directly bind to promoters of BCL-2, BSP, NANOG, XIAP, KLF4, and HOXA2, suggesting SATB2 is capable of directly regulating pluripotency/self-renewal, cell survival, and proliferation. Overall, our data suggest that the SATB2 gene acts as an oncogene in prostate cancer, and its expression alone is capable of inducing oncogenic transformation in vitro.

Yamanaka and colleagues were able to generate iPSC by overexpressing four factors, namely OCT4, SOX2, KLF4, and cMYC, in fibroblasts [49]. The trans-differentiation technique has allowed us to generate desired cell types using iPS [50,51]. The use of iPS has been very useful in the area of medicine by generating specialized cell types [50,51]. Expression of lineage-specific regulatory genes can promote direct conversion or trans-differentiation from one mature differentiated cell type into a distinct differentiated cell type [52,53,54,55]. Thus, pluripotency factors can induce an epigenetically unstable state that is responsive to environmental signals which allows direct conversion of stem cells to lineage-specific progenitors and differentiated derivatives. These differentiated cells are capable of performing specialized functions.

SATB2 promoter contains NANOG, cMYC, and OCT4 binding sites [56,57,58]. NANOG, OCT4, cMYC, SOX2, and KLF4 genes contain SATB2 binding sites. These predicted models suggest that NANOG, cMYC, and OCT4 can regulate SATB2; and SATB2 can regulate NANOG, OCT4, cMYC, SOX2, and KLF4. Thus, regulation of SATB2 can modulate the expression of pluripotency-maintaining and self-renewal factors. Since SATB2 binding sites are present in the promoters of NANOG, OCT4, cMYC, SOX2, and KLF4 (pluripotency genes), SATB2 can act as a biological regulator of pluripotency and self-renewal of CSCs. In the present study, SATB2 is expressed in transformed cells, but not in normal prostate tissues, suggesting its use as a biomarker of prostate cancer. Similarly, we have demonstrated that SATB2 can induce cellular transformation of epithelial cells derived from normal pancreatic, colorectal, and breast tissues [31,32]. Overall, these studies will enhance our understanding of the role of SATB2 in prostate carcinogenesis.

Our ChIP data also showed that SATB2 can bind to the promoters of XIAP, NOXA2, BCL-2, and BSP genes. XIAP blocks apoptosis by blocking caspase activation [59]. NOXA2 improves erectile dysfunction in rats with type I diabetes by inhibiting oxidative stress and corporal fibrosis [60]. Pagala et al. reported that NOXA2 may protect proteins involved in electron transfer by reducing oxygen to hydrogen peroxide [61]. BCL-2 rinhibits apoptosis and upregulates cell proliferation through multiple mechanisms [62,63]. Apoptosis disruption may result in several diseases, including cancer associated with the inhibition of apoptosis and neurodegenerative disorders associated with the enhancement of apoptosis [63,64,65]. Bone sialoprotein (BSP) is an extracellular matrix protein found in mineralized tissues of the skeleton and dentition [66,67]. BSP is multifunctional, affecting cell attachment and signaling through an Arg-Gly-Asp integrin-binding region, and is involved in cementoblast differentiation and/or early mineralization of the cementum matrix [68,69]. These findings suggest that SATB2 can regulate various cellular functions related to pluripotency, self-renewal, proliferation, and survival. 

The process of EMT allows cells to acquire a new phenotype, leave the primary site of origin, become invasive, and migrate and invade distant sites [40]. This process of EMT is mediated by a group of transcription factors (ZEB1, SNAIL, and SLUG) which negatively regulate the expression of E-CADHERIN, an epithelial protein. In the present study, SATB2 overexpression in PrECs promoted EMT, and its inhibition in prostate cancer stem cells (CSCs) suppressed/reversed EMT characteristics. Specifically, SATB2 shRNA inhibited the expression of SNAIL, SLUG, ZEB1, and N-CADHERIN, and induced the expression of E-CADHERIN, a characteristic generally observed in MET. Similarly, in other models of cancer, we have demonstrated that overexpression of SATB2 induced EMT, and SATB2 knockdown in CSCs inhibited EMT characteristics [31,32]. These data suggest that SATB2 has the potential to enhance EMT and metastasis.

In the present study, human prostate adenosarcoma tissues expressed higher SATB2 than adjacent normal tissues. Using receiver operator characteristic analysis, SATB2 was a sensitive and specific marker for prostate adenocarcinoma (100.0%, 80.0%). Similarly, we have previously reported the higher expression of SATB2 in breast, pancreatic, liver, and colorectal cancer [32,33,37]. In another study, Hoskoppal et al. found that SATB2 protein expression was a sensitive and specific marker of appendiceal and rectosigmoid well-differentiated neuroendocrine tumors [70]. Based on these data, SATB2 can be used as a biomarker and therapeutic target for cancer [22].

## 5. Conclusions

In conclusion, our data demonstrate that overexpression of SATB2 in PrECs induces malignant transformation in vitro, and those transformed cells gained the properties of CSCs. We also demonstrate that SATB2 is not expressed in normal PrECs, but it is highly expressed in CSCs, cancer cells, and primary tumor tissues. SATB2 knockdown in prostate CSCs suppresses spheroid formation, cell viability, colony formation, cell motility, migration, and invasion. In addition, SATB2 can directly bind to the promoters of BCL-2, XIAP, BSP, KLF4, cMYC, HOXA2, and Nanog genes. Further studies are needed to examine the oncogenic role of SATB2 in a transgenic mouse model of prostate cancer. 

## Data Availability

The data presented in this study are available on request from the corresponding author.
